# Effect of westernization on oral health among college students of Udaipur City, India

**DOI:** 10.1186/s40779-016-0103-4

**Published:** 2016-10-26

**Authors:** Piyush Pujara, Neeraj Sharma, Rujul Jayeshkumar Parikh, Maitri Shah, Shachi Parikh, Vivek Vadera, Manpreet Kaur, Isha Makkar, Mayur Parmar, Pratik Rupakar, Shrikant Patel

**Affiliations:** 1 Department of Public Health Dentistry, Pacific Dental College and Hospital, Airport Road, Debari, Udaipur, Rajasthan India; 2 Department of Prosthodontics, Modern Dental College and Research Centre, Indore, India; 3 Dr.D.y. Patil Dental College and Hospital, Pune, India; 4 Faculty of Dental Science, Ndaiad, Gujarat India; 5 Department of Oral Medicine, Karnavati School of Dentistry, Ahmedabad, India; 6 Pacific Dental College and Hospital, Udaipur, India; 7 Pacific Medical College and Hospital, Udaipur, India; 8 Senior Lecturer, Department of Periodontics, Goenka Research Institute of Dental Science, Piplaj Gandhinagar, India; 9 Department of Dentistry, Gujarat Adani Institute of Medical Science, Bhuj, Kutch, Gujarat India; 10 Senior Lecturer, Department of Oral Pathology, Pacific Dental College and Hospital, Udaipur, India

**Keywords:** Westernization, Periodontal disease, Dental caries, Food

## Abstract

**Background:**

There is overwhelming evidence that periodontal disease and dental caries affect the majority of populations and that western culture and lifestyle may have a profound influence on oral health, especially in adults. The present study was performed to determine the effect of westernization on the oral health of college students of Udaipur City, Rajasthan.

**Methods:**

A descriptive cross-sectional study was conducted among students attending various professional and non-professional bachelor’s degree colleges of Udaipur City, Rajasthan, India, from March 2013 to May 2013. Eight hundred students were selected based on a two-stage random sampling procedure. Westernization was assessed by a self-administered structured questionnaire. Periodontal status, dental caries status and malocclusion were assessed according to World Health Organization (WHO) criteria (1997). Statistical analysis was performed using chi-square and Multivariate logistic regression. The confidence level and level of significance were set at 95 and 5 %, respectively.

**Results:**

The present study suggested that adverse habits, listening to English music and preferring English food had a significant association with dental caries and periodontal diseases. Malocclusion also showed a significant relationship with consuming English food for snacks and desserts. Multivariate analysis revealed a significantly greater odds ratio (*OR*) for periodontal disease and dental caries among those who preferred English food for lunch.

**Conclusion:**

Based on the results of the present study, there is an association between westernization and oral health.

## Background

India, similar to many emerging economies worldwide, is in the midst of an epidemiologic transition that is characterized by rising rates of chronic disease and is driven largely by modifiable behavioural risk factors [1]. Westernization is a process whereby societies come under or adopt Western culture in areas such as industry, technology, law, politics, economics, lifestyle, diet, clothing, language, alphabet, religion, philosophy, and values [2]. Westernization has been an accelerating influence across the world over the last few centuries, with some researchers assuming westernization to be the equivalent of modernization [3], a way of thought that is often debated. This transition appears to be fuelled by increasing urbanization, industrialization, and globalization. These factors are changing the way people live and the environments in which they reside in ways that may escalate behavioural risk. Globalization -- the increasing interconnectedness of countries and the openness of borders to ideas, people, commerce, and financial capital -- has beneficial and harmful effects on the health and oral health of populations [4, 5]. Although the effects of urbanization and industrialization on chronic disease and its related behavioural risk factors are well documented, the effects of globalization are less well specified [1].

Exposure to different types of media, such as music, movies, television, and the Internet, facilitates the exchange of global ideas and information, thus making subsequent changes in beliefs and behaviours possible, especially for adults [6]. This process by which individuals adopt the attitudes, values, customs, beliefs, and behaviours of another culture is called acculturation. Acculturation entails the social and psychological exchanges that occur when there is continuous contact and interaction between individuals from different cultures [7–9]. Studies have shown that high levels of acculturation among Latinos are associated with increased rates of cancer, infant mortality, and other indicators of poor physical and mental health [10]. With some exceptions, the rates of risky health behaviours (e.g., smoking, alcohol use, high body mass index) also increase with acculturation [11]. In contemporary India, the most pervasive influence on what defines “global culture” is that of the West, especially that from the United States and the United Kingdom. English movies, music, and television shows are prominent and popular here, as are Western goods, such as food, soft drinks, and clothing. The increased consumption of junk foods, carbonated beverages, sweets, tobacco and alcohol is associated with westernization. Fast foods are a wide range of easily prepared meals and have gained in popularity with the rise of the increasingly hectic modern lifestyle [12]. Food courts, shopping malls and brands of cigarettes are popular with youth in the West [13–15]. In response to this growing globalization, market researchers and advertising agencies in India now commonly craft hybrid images and messages that reflect this new bi-cultural identity – one that is both local (i.e., Indian) and global (i.e., Western) [16].

According to the surgeon general’s report on “oral health in America”, diet and nutrition are major multi-factorial environmental factors in the aetiology and pathogenesis of craniofacial diseases and disorders [17]. Sugar in the diet and bacterial dental plaque are important etiological factors for dental caries and periodontal diseases [18]. Oral cancer is a debilitating disease that affects a minority of the population, but its prevalence is increasing because of risk factors such as tobacco smoking and alcohol consumption [19]. It is also evident that oral diseases result in impairment and physical disabilities that impact the social, emotional and psychological aspects of life. There is overwhelming evidence that periodontal disease and dental caries affect the majority of populations [6] and that western culture and lifestyle may have a profound influence on oral health, especially in adults.

Over the last several years, with the development of malls, multiplexes, and tourism in Udaipur City, India, western culture is creeping into the daily lives of adults. Although the body of literature on the effect of globalization on health is growing [20], no commonly accepted theoretical framework exists to describe and explain the direct and indirect effects that diverse aspects of globalization can have on oral health [4]. Therefore, the present study was undertaken with the objective of determining the association of westernization with oral health among college going students of Udaipur City, Rajasthan.

## Methods

### Study design, study setting and study population

A descriptive cross-sectional study was conducted among students attending various professional and non-professional bachelor’s degree colleges of Udaipur City, Rajasthan, India, from March 2013 to May 2013. The present study sought to determine the effect of westernization parameters such as eating English food and listening to English music on oral health indicators such as dental caries and periodontal disease.

### Ethical approval and official permission

Official permission was obtained from the respective principals of the involved colleges.

### Informed consent

Following the investigators’ explanation of the purpose and details of the study, written informed consent was obtained from all participants.

### Training and calibration

Before the commencement of the study, the examiner was standardized and calibrated in the Department of Public Health Dentistry by a senior Faculty member [Professor and Head] to ensure uniform interpretation, understanding and application of the codes and criteria for the diseases to be observed and recorded and to ensure consistent examination.

### Inclusion criteria

1. Students attending various colleges (professional and non-professional) of Udaipur City available during the study period; 2. Students who were intellectually and physically capable of responding to the questionnaire; 3. Students who were willing to participate in the study; 4. Students who exhibited smoking and poor brushing habits.

### Exclusion criteria

1. Students attending dental colleges of Udaipur City; 2. Students who were not willing to participate; 3. Students undergoing orthodontic treatment; and 4. Students suffering from any systemic diseases.

### Pilot study and pre-testing of the questionnaire

A self-administered structured questionnaire was developed and administered to a convenience sample of 50 students who were interviewed to gain feedback on the overall acceptability of the questionnaire in terms of length and language clarity. Based on their feedback, the questionnaire did not require any correction. Cronbach’s coefficient was found to be 0.78, which showed the internal reliability of the questionnaire. The mean content validity ratio (CVR) was calculated as 0.87, based on the opinions expressed by a panel of four academicians. Face validity was also assessed, and it was observed that 92 % of the participants found the questionnaire to be easy. Based on the results of the pilot study and a 95 % confidence level, 5 % precision and 80 % power, the final sample size was 800.

### Proforma details

A structured 24-item questionnaire was designed to ascertain the effect of westernization on oral health. The questionnaire included details on gender, socioeconomic status and adverse habits. Four domains of culture were measured to assess westernization: preferences for (a) language; (b) media; (c) food; and (d) consumer goods. The list of questions appears in Table 1, which is organized by dimensions and domains. The responses to each item were on a four-point Likert scale: (1) never, (2) sometimes, (3) often, or (4) very often.

**Table 1:** Distribution of four domains of the western scale [*n* (%)]

Western scale	Never	Sometimes	Often	Very often
Language preference				
Speak with mother or father in English	648 (81.0)	80 (10.0)	48 (6.0)	24 (3.0)
Speak with siblings in English	437 (54.6)	308 (38.5)	7 (0.9)	48 (6.0)
Speak with friends in English	432 (54.0)	304 (38.0)	2 (0.2)	62 (7.7)
Media preference				
Watch English TV shows	192 (24.0)	156 (19.5)	168 (21.0)	284 (35.5)
Watch English movies or films	88 (11.0)	192 (24.0)	412 (51.5)	108 (13.5)
Listen to English music	129 (16.1)	90 (11.3)	474 (59.3)	107 (13.4)
Preference for food				
Eat English food for lunch	80 (10.0)	104 (13.0)	184 (23.0)	432 (54.0)
Eat English food for a snack	88 (11.0)	88 (11.0)	360 (45.o)	264 (33.0)
Eat English food for dessert	106 (13.3)	133 (16.6)	290 (36.3)	271 (33.9)
Other consumer goods				
Wear Western clothing	192 (24.0)	311 (38.9)	213 (26.6)	84 (10.5)
Go to a Western coffee house	84 (10.5)	172 (21.5)	280 (27.0)	264 (42.0)
Go to a Western-type shopping mall	76 (2.5)	172 (9.5)	216 (31.0)	336 (57.0)

### Oral health status

The oral health status of students was measured according to WHO guidelines [21] using the following indices: (1) Community periodontal index and loss of attachment; (2) Dentition status and treatment needs; and (3) Dental aesthetic index (DAI).

### Sampling design

Before beginning the study, the official list of all colleges in Udaipur City was obtained from Mohanlal Sukhadia University, Udaipur, Rajasthan. A two-stage random sampling procedure was used to select the study sample. In the first stage, 20 % (20 colleges) of all colleges (102) were randomly selected using the lottery method. The second stage was to select students from each selected college. Lists of students enrolled in the 20 selected colleges were obtained. From a list encompassing all of the students (9,996) enrolled in the 20 colleges, a sample of 800 students was selected based on a systematic random sampling procedure.

### Data collection

The examiner visited the colleges on the predetermined dates according to the schedule. Based on the examiner’s instructions, subjects were asked to complete the questionnaire. Furthermore, they were clinically examined (Type III examination) according to the WHO guidelines [21] on a proforma attachment to their respective questionnaires.

### Statistical analysis

The data were coded and entered into a Microsoft Excel spread sheet. Analysis was performed using SPSS version 15.0 for Windows. Descriptive statistics included the computation of percentages. The statistical tests applied for the analysis were Pearson’s chi-square test and multivariate logistic regression analysis. For all tests, the confidence level and level of significance were set at 95 and 5 %, respectively.

## Results

### Distribution of four domains of the western scale

Among all subjects, there were 435 (54.4 %) males and 365 (45.6 %) females. Nearly half of participants belonged to the middle socioeconomic group [*n* = 398 (49.8 %)] and had one adverse habit [*n* = 368 (46 %)]. Overall, 81 % participants never spoke in English with their mother and father. In the media preference domain, 35.5 % subjects watched English TV shows very often, 51.5 % watched English movies and 49.3 % listened to English music often. Approximately 54.0, 33.0 and 33.9 % of subjects preferred English food for lunch, snacks and desserts very often, respectively (Table 1).

### Periodontal status

Overall, participants with adverse habits had significantly higher (*P* = 0.001) rates of periodontal disease (66.6 %) than their counterparts. A greater proportion (18.4 %) of subjects who listened to English music had periodontal disease in compared with that of subjects who watched English TV (14.8 %) and watched English movies (14.8 %) of films, but there is no statistical difference among three groups. Those who listen to English was significantly correlated with CPI index (Table 2). Multivariate analysis revealed a significantly greater odds ratio for periodontal disease among those who preferred English food for lunch (*OR* = 3.5, *P* = 0.001) and snacks (*OR* = 1.4, *P* = 0.01, Table 3).

**Table 2:** Associations of periodontal status (CPI) with several independent variables

Variables	Community Periodontal Index scores [*n* (%)]					
	Healthy	Bleeding	Calculus	Pocket (4–5 mm)	Pocket (6 mm or more)	*P* value
Gender						0.24
Male (*n* = 435)	107 (24.6)	128 (29.4)	124 (28.5)	66 (15.2)	10 (2.3)	
Female (*n* = 365)	74 (20.3)	101 (27.7)	118 (32.3)	56 (15.3)	16 (4.4)	
Socioeconomic status						0.75
Low (*n* = 190)	49 (25.8)	49 (25.8)	58 (30.5)	28 (14.7)	6 (3.2)	
Medium (*n* = 398)	89 (22.4)	120 (30.2)	113 (28.4)	65 (16.3)	11 (2.8)	
High (*n* = 212)	43 (20.3)	60 (28.3)	71 (33.5)	29 (143.7)	9 (4.2)	
Adverse habits						0.001^*^
Present (*n* = 368)	123 (33.4)	122 (33.2)	87 (23.6)	28 (7.6)	8 (2.2)	
Absent (*n* = 432)	58 (13.4)	107 (24.8)	155 (35.9)	94 (21.8)	18 (4.2)	
Western scale						
Language preference						
Speak with mother or father in English (*n* = 72)	17 (23.6)	29 (40.3)	14 (19.4)	9 (12.5)	3 (4.2)	0.11
Speak with siblings in English (*n* = 55)	33 (60.0)	9 (14.5)	8 (7.3)	4 (16.4)	1 (1.8)	0.21
Speak with friends in English (*n* = 64)	30 (46.9)	19 (29.7)	9 (14.1)	6 (9.37)	0	0.35
Media preference						
Watch English TV shows (*n* = 452)	122 (27.0)	118 (260.1)	130 (28.8)	67 (14.8)	15 (3.3)	0.10
Watch English movies or films (*n* = 520)	134 (25.8)	148 (28.5)	143 (27.5)	77 (14.8)	18 (3.5)	0.07
Listen to English music (*n* = 581)	135 (23.2)	149 (25.6)	170 (29.3)	107 (18.4)	20 (3.4)	0.03^*^
Preference for food						
Eat English food for lunch (*n* = 616)	151 (24.5)	200 (32.5)	166 (26.9)	85 (13.8)	14 (2.3)	0.001^*^
Eat English food for a snack (*n* = 624)	141 (22.6)	174 (27.9)	188 (30.1)	98 (15.7)	23 (3.7)	0.001^*^
Eat English food for dessert (*n* = 561)	124 (22.1)	167 (29.8)	163 (29.1)	84 (15.0)	23 (4.1)	0.04^*^
Other consumer goods						
Wear Western clothing (*n* = 297)	79 (26.6)	91 (30.6)	85 (28.6)	37 (12.5)	5 (1.7)	0.23
Go to a Western coffee house (544)	25 (23.0)	142 (26.1)	169 (31.1)	87 (16.0)	21 (3.9)	0.05
Go to a Western-type shopping mall (*n* = 552)	130 (23.6)	154 (27.9)	161 (29.2)	84 (15.2)	23 (4.2)	0.17

Test applied Chi-square test, ^*^statistically significant at *P* < 0.05

**Table 3:** Multivariate logistic regression considering the association between the dependent variable (CPI) and independent variables

Clinical indicators	Adjusted *OR*	*P*-value	95 % confidence interval
Male /Female	0.96	0.95	0.68–1.43
Listen to English music (Yes/No)	0.31	0.001^*^	2.07–5.92
Eat English food for lunch (Yes/No)	3.50	0.001^*^	0.19–0.49
Eat English food for a snack (Yes/No)	1.40	0.01^*^	0.54–0.82
Eat English food for dessert (Yes/No)	1.43	0.11	0.91–2.32
Go to a western coffee house (Yes/No)	1.40	0.10	0.93–2.11

^*^Statistically significant at *P* < 0.05

### Dental caries

Dental caries was found to be significantly (*P* = 0.001) less among those with adverse habits (82.3 %) than their counterparts (83.5 %). In the domain of media preference, those watching English movies and listening to English music showed a significant association with dental caries (Table 4). Multivariate analysis revealed a significantly greater odds ratio for dental caries among those who preferred English food for lunch (*OR* = 2.70, *P* = 0.03, Table 5).

**Table 4:** Associations of dental caries status with various independent variables [*n* (%)]

Variables	Dental caries status				
	None (0)	Mild (0.1–2.6)	Moderate (2.7–4.5)	Severe (>4.5)	*P* value
Gender					
Male (*n* = 435)	74 (17.01)	122 (28.1)	147 (33.9)	92 (21.2)	0.61
Female (*n* = 365)	64 (17.53)	115 (31.6)	109 (29.9)	77 (21.2)	
Socioeconomic status					
Low (*n* = 190)	26 (13.6)	47 (24.7)	64 (33.6)	53 (27.8)	0.11
Medium (*n* = 398)	75 (18.8)	124 (31.2)	126 (31.6)	73 (18.3)	
High (*n* = 212)	36 (17.1)	66 (31.3)	66 (31.3)	43 (20.4)	
Adverse habits					
Present (*n* = 368)	65 (17.7)	143 (39.0)	82 (22.3)	77 (21.0)	0.001^*^
Absent (*n* = 432)	71 (16.5)	94 (21.8)	174 (40.4)	92 (21.3)	
Western scale					
Language preference					
Speak with mother or father in English (*n* = 72)	5 (6.9)	30 (41.7)	26 (36.1)	11 (15.3)	0.34
Speak with siblings in English (*n* = 55)	0	22 (40.0)	26 (47.3)	7 (12.7)	0.12
Speak with friends in English (*n* = 63)	11 (17.5)	31 (49.2)	7 (11.1)	14 (22.2)	0.21
Media preference					
Watch English TV shows (*n* = 451)	40 (8.9)	156 (34.6)	163 (36.1)	92 (20.4)	0.43
Watch English movies or films (*n* = 519)	74 (14.3)	178 (34.3)	177 (34.1)	90 (17.3)	0.01^*^
Listen to English music (*n* = 579)	84 (14.5)	214 (37.0)	167 (28.8)	114 (19.7)	0.02^*^
Preference for food					
Eat English food for lunch (*n* = 614)	100 (16.3)	214 (34.9)	165 (26.9)	135 (22.0)	0.001^*^
Eat English food for a snack (*n* = 623)	104 (16.7)	194 (31.1)	190 (30.5)	135 (21.7)	0.001^*^
Eat English food for dessert (561)	95 (16.9)	181 (32.3)	177 (31.6)	108 (19.3)	0.001^*^
Other consumer goods					
Wear Western clothing (*n* = 296)	52 (17.6)	103 (34.8)	84 (28.4)	57 (19.3)	0.07
Go to a Western coffee house (*n* = 542)	82 (15.1)	160 (29.5)	167 (30.8)	133 (24.5)	0.003^*^
Go to a Western-type shopping mall (*n* = 551)	98 (17.8)	199 (36.1)	163 (29.6)	91 (16.5)	0.17

Chi-square test, ^*^ statistically significant at *P* < 0.05

**Table 5:** Multivariate logistic regression considering the association between the dependent variable (DMFT) and independent variables

Clinical indicators	Adjusted *OR*	*P*- value	95 % CI
Male /female	0.84	0.48	0.52–1.35
Listen to English music (Yes/No)	0.66	0.15	0.38–1.16
Watch English movie (Yes/No)	0.92	0.02^*^	1.08–2.76
Eat English food for lunch (Yes/No)	2.70	0.03^*^	1.61–4.55

^*^Statistically significant at *P* < 0.05

### DAI

Only the preference for English food for snacks and dessert showed a significant association with DAI (Table 6).

**Table 6:** Associations of DAI scores with various independent variables [*n* (%)]

Variables	DAI				
	No abnormality or minor malocclusion (No/slight need)	Definite malocclusion (Elective treatment)	Severe malocclusion (Highly desirable)	Very severe or handicapping malocclusion (Mandatory)	*P* value
Gender					0.12
Male (*n* = 435)	314 (72.2)	54 (12.4)	53 (12.2)	14 (3.2)	
Female (*n* = 365)	257 (70.4)	54 (14.8)	43 (11.8)	11 (3.0)	
Socioeconomic status					0.69
Low (*n* = 190)	134 (70.5)	28 (14.7)	20 (10.5)	8 (4.2)	
Medium (*n* = 398)	283 (71.1)	49 (12.3)	53 (13.3)	13 (3.3)	
High (*n* = 212)	154 (72.6)	31 (14.6)	23 (10.8)	4 (1.9)	
Adverse habits					0.46
Present (*n* = 368)	259 (70.4)	53 (13.0)	48 (14.4)	8 (2.2)	
Absent (*n* = 432)	312 (72.2)	60 (13.9)	43 (10)	17 (3.9)	
Western scale					
Language preference					
Speak with mother or father in English (*n* = 72)	60 (83.3)	11 (15.2)	1 (1.3)	0	0.56
Speak with siblings in English (*n* = 55)	45 (81.8)	3 (5.5)	4 (7.3)	3 (5.5)	0.23
Speak with friends in English (*n* = 64)	51 (79.7)	13 (20.3)	0	0	0.45
Media preference					
Watch English TV shows (*n* = 452)	316 (69.9)	51 (11.3)	72 (15.9)	13 (2.9)	0.16
Watch English movies or films (*n* = 520)	358 (68.8)	59 (11.3)	87 (16.7)	16 (3.1)	0.12
Listen to English music (*n* = 581)	387 (66.6)	93 (16.0)	79 (13.6)	22 (3.8)	0.09
Preference for food					
Eat English food for lunch (*n* = 616)	438 (71.1)	83 (13.5)	85 (13.8	10 (1.8)	0.17
Eat English food for a snack (*n* = 624)	431 (69.1)	88 (14.1)	81 (13.5)	24 (3.4)	0.05
Eat English food for dessert (*n* = 561)	421 (75.0)	79 (8.2)	46 (14.1)	15 (2.7)	0.001^*^
Other consumer goods					
Wear Western clothing (*n* = 297)	209 (70.4)	41 (13.8)	40 (13.5)	7 (2.4)	0.41
Go to a Western coffee house (*n* = 544)	371 (68.2)	83 (15.3)	75 (13.8)	15 (2.8)	0.90
Go to a Western-type shopping mall (*n* = 552)	408 (73.9)	78 (14.1)	52 (9.4)	14 (2.5)	0.76

Test applied Chi-square test, ^*^statistically significant at *P* < 0.05

## Discussion

Globalization is one of the key challenges that face health policymakers and public health practitioners [22]. Although many studies have addressed the importance of globalization in health [23, 24], there is no consensus on the pathways and mechanisms through which globalization affects the oral health of populations or the appropriate policy responses. The present study aimed to evaluate the effects of westernization on the oral health of college students of Udaipur City, Rajasthan. In total, eight hundred college students between the ages of 19 and 25 years were evaluated.

A self-administered structured questionnaire was designed to evaluate the effect of westernization on oral health. Four domains of culture were measured: preferences for (a) language; (b) media; (c) food; and (d) consumer goods. The present study suggested that English food and desserts had a significant association with caries and periodontal diseases. Similar results were observed by Sakki et al. [25] and Liu [26], who showed that an unhealthy diet was associated with a higher prevalence of dental caries and periodontal pocketing. According to the surgeon general’s report on oral health in America, diet and nutrition are major multi-factorial environmental factors in the aetiology and pathogenesis of craniofacial diseases and disorders [17].

Adverse habits also showed a significant relationship with dental caries and periodontal disease in the present study sample, which was comparable with the results of Hessari et al. [27] among middle-aged adults, particularly in males, in whom smoking and lifelong exposure to smoking with a dose-dependent effect seem to be associated with higher periodontal treatment needs and poorer dental status.

Another interesting finding of the present study was that those who listened to English music, watched English movies, and went to Western coffee houses often had more dental diseases than did their counterparts, which was in agreement with the study conducted by Petersen & Nortov [28], who found that people who watched TV, visited coffee houses and listened to the radio more often had more symptoms in their teeth and gums and visited a dentist more frequently. In the present study, gender and socioeconomic status did not show any significant relationship with dental diseases, which was in agreement with the findings of Paula et al. [29] and Acevedo et al. [30].

Malocclusion also showed a significant relationship with participants who preferred eating English food for snacks and dessert. This may be because English food is generally sticky, which lead to extensive untreated carious lesions and, subsequently, high levels of premature tooth loss in children, resulting in malocclusion.

The present study highlights the significant association between western culture adopted by adults and oral diseases such as dental caries, periodontitis and malocclusion. However, the study did not identify the exact mechanisms by which westernization affects oral health among young people. One important point to consider is that oral hygiene has a larger impact on dental disease than eating English food or listening to English music. An important limitation to consider is that our measure of westernization is behaviourally based. Therefore, further investigations are recommended that might address the role of oral health care practitioners in emphasizing prevention and lifestyle changes that benefit oral health.

## Conclusion

Based on the results of the present study, there is an association between westernization and oral health. This suggests that young people’s identification with western culture may increase their risk for oral diseases. Therefore, lifestyle counselling, especially in high-risk groups, may help improve the oral health of adults.

## Abbreviations

CVR: Content validity ratio
DAI: Dental aesthetic index
OR: Odds ratio
WHO: World Health Organization
